# Bispecific Antibody and Antibody-Drug Conjugate as Novel Candidates for Treating Pancreatic Ductal Adenocarcinoma

**DOI:** 10.3390/biom15101477

**Published:** 2025-10-20

**Authors:** Hyeryeon Seo, Dabin Go, Se Young Jung, Shinwoo Han, Van Quy Nguyen, Minseok Kwak, Wooram Um

**Affiliations:** 1 Department of Biotechnology, Pukyong National University, Busan 48513, Republic of Korea; 2 Faculty of Chemical and Food Technology, University of Technology and Education, Ho Chi Minh City 75603, Vietnam; 3 Department of Chemistry and Industry 4.0 Convergence Bionics Engineering, Pukyong National University, Busan 48513, Republic of Korea

**Keywords:** pancreatic ductal adenocarcinoma, bispecific antibody, antibody-drug conjugate

## Abstract

Pancreatic ductal adenocarcinoma (PDAC) is one of the most lethal cancers, characterized by a dense and immunosuppressive tumor microenvironment. With the limited actions of drugs and conventional monovalent antibodies, the success of existing cancer therapies is restricted so far. Recently, bispecific antibodies (BsAbs) have emerged as a promising therapeutic platform, capable of overcoming the limitations of current PDAC treatments by engaging T cells and delivering drugs to multiple targets in a selective manner. Furthermore, the recruitment of additional payloads expands their therapeutic potential, offering more selective drug delivery and presenting new possibilities for treating PDAC. However, a limited number of relevant studies and a lack of comprehensive research have hindered trials for the development of BsAbs and bispecific antibody-drug conjugates (BsADCs) in PDAC therapeutics. This review aims to provide the characteristics of BsAbs and BsADCs and their recent applications in PDAC treatment. Additionally, frequent targets of PDAC treatments will be discussed to suggest how to design BsAbs and BsADCs for PDAC treatments.

## 1. Introduction

Pancreatic ductal adenocarcinoma (PDAC) is one of the most lethal malignancies, with a dismal prognosis despite decades of clinical efforts. Particularly, PDAC is the most common type of pancreatic cancer, accounting for more than 90% of cases [1,2]. According to recent Surveillance, Epidemiology, and End Results (SEER) data, the 5-year relative survival rate of PDAC remains approximately 12–13% in the United States, far below the average of other types of cancers [3]. Primarily, it might be attributed to the difficulty of early detection of PDAC, since early symptoms in PDAC are either absent or nonspecific. Therefore, over 80% of patients are diagnosed at an advanced or metastatic stage when surgical resection is no longer feasible. Additionally, the majority of patients are considered to be surgically unresectable due to complicated vascular involvement and the high dependency of PDAC in cases of locally advanced growth. Therefore, even among the 20% or fewer patients who undergo resection, recurrence rates remain high, and 5-year survival rarely exceeds 30% [4]. Thus, anti-cancer treatments are more important for PDAC than other types of cancer.

Despite substantial efforts in PDAC treatments, the clinical outcomes of conventional therapies for PDAC have been disappointing thus far due to its unique tumor microenvironment (TME). Molecularly, pancreatic cancer is characterized by nearly universal Kirsten Rat Sarcoma Viral Oncogene Homolog (KRAS) mutations (>90%), and high expressions of multidrug resistance genes. These features collectively limit the efficacy of conventional chemotherapies. For example, standard chemotherapy regimens such as FOLFIRINOX or gemcitabine with nab-paclitaxel extend the median survival to only 10–12 months in PDAC [5]. Furthermore, desmoplastic stroma and poorly organized tumor vasculature have also remained significant challenges in the treatment of PDAC. These abnormal anatomical features obstruct the diffusion of oxygen and drugs and impede the infiltration of immune cells, resulting in poor drug delivery efficiency and immune responses. Therefore, immune checkpoint inhibitors (anti-PD-1/PD-L1, anti-CTLA-4) have also shown minimal benefit in pancreatic cancer due to the highly immunosuppressive TME [6]. As a result, pancreatic cancer remains a “silent killer” with significant therapeutic resistance and unmet medical needs.

Bispecific antibodies (BsAbs) have recently been developed as next-generation agents for treating refractory cancers. BsAbs are engineered immunoglobulins that can bind to two different targets or epitopes simultaneously. By targeting two mechanisms concurrently, BsAbs can enhance tumor selectivity and reduce the likelihood of tumor escape or therapeutic resistance. For example, T-cell-engaging BsAbs not only can redirect cytotoxic lymphocytes to attack tumor cells but also reprogram the immunosuppressive TME by using the opposite side [7]. It is noteworthy that BsAbs offer diverse therapeutic approaches unattainable with conventional chemotherapeutic drugs or antibodies.

Similarly, Bispecific antibody–drug conjugates (BsADCs) represent an innovative extension of BsAbs, combining multiple binding affinities with potent cytotoxic payloads. The primary premise of BsADCs is that co-recognition of two antigens can enable more selective drug delivery and uptake in tumor cells, mitigating the off-tumor toxicities seen with traditional single-target ADCs [8]. In other words, a BsADC is designed to exert cytotoxic effects predominantly on cells co-expressing both target antigens, thereby sparing normal tissues that express only one [9]. Collectively, these advances underscore the potential of BsAbs and BsADCs to surmount clinical and molecular challenges of PDAC, and they provide a strong rationale for pursuing these approaches as next-generation strategies to improve outcomes in treating PDAC [10,11]. However, the applicability of BsADCs for the treatment of PDAC has not been the focus of much discussion to date, due to the limited literature so far. In this research, we thus comprehensively review the definition, structure, and mechanisms of action of BsAbs and BsADCs for treating PDAC. Furthermore, we evaluate their preclinical and clinical applications in PDAC and discuss their key achievements and remaining limitations. Finally, we discuss how these platforms can address unmet clinical needs in pancreatic cancer treatment and propose future directions [10,11].

## 2. BsAbs and Their Therapeutic Potential in PDAC

### 2.1. Classification of BsAbs

**Figure 1:**
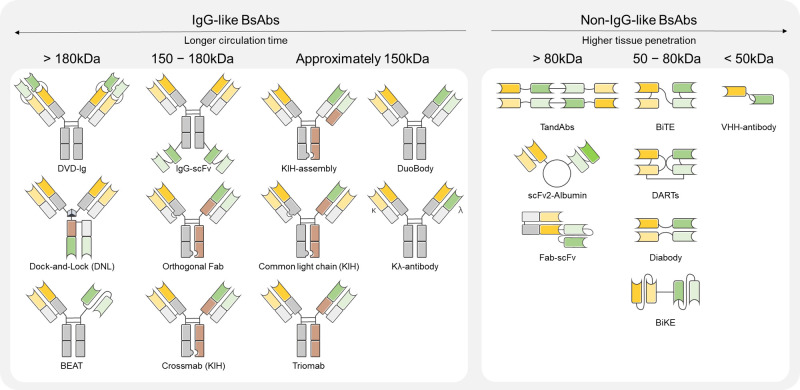
Types of BsAbs by molecular weights.

BsAbs are engineered proteins that recognize two distinct antigens or epitopes within a single molecule. This dual recognition enhances therapeutic specificity and efficacy in oncology and immune-mediated diseases. Unlike monoclonal antibodies (mAbs), which act on a single target, BsAbs provide dual targeting capabilities, enabling either complex mechanisms of action or direct immune cell redirection, thus positioning them as a promising next-generation therapeutic strategy [10]. The structure of BsAbs dictates their mechanisms of action. IgG-like BsAbs are suited for effector-mediated functions and dual-receptor blockade, whereas non-IgG-like BsAbs are optimized for T-cell-mediated immune activation. Based on their structural framework, BsAbs are broadly categorized into IgG-like and non-IgG-like types as shown in Figure 1. Due to their structural properties, there are significant differences between BsAbs in terms of immune activation, serum half-life and tissue penetration [12].

#### 2.1.1. IgG-like BsAbs

IgG-like BsAbs retain the conventional IgG backbone and incorporate the Fc domain, facilitating effector functions such as antibody-dependent cellular cytotoxicity (ADCC) and complement-dependent cytotoxicity (CDC) through interaction with Fc receptors [12]. This architecture is advantageous for enhancing serum half-life and ensuring molecular stability as a therapeutic antibody. A representative platform within this category is the Knobs-into-Holes (KIH) technology, developed to prevent nonspecific heavy chain pairing by promoting selective heterodimerization of the Fc region. Amivantamab (EGFR×MET), a KIH-based BsAb, has shown efficacy in non-small-cell lung cancer (NSCLC) with EGFR mutations and MET amplification by inhibiting both pathways. It acts via multiple mechanisms, including Fc-mediated ADCC and receptor internalization [13]. Additionally, it has been reported to function via Fc-independent mechanisms, effectively inducing therapeutic responses even under ligand-dependent TME [14]. On the other hand, CrossMab technology resolves heavy/light chain mispairing by domain crossover to ensure stable bi-specificity [12]. Faricimab (VEGF-A×Ang-2) outperformed VEGF monotherapy by extending treatment intervals while maintaining vision [15,16]. Other IgG-like platforms include Duobody and Dual-Variable Domain Immunoglobulin (DVD-Ig), both of which are engineered to enhance specificity while maintaining the favorable pharmacological properties of classical IgG structures. Duobody antibodies are generated via controlled Fab-arm exchange by recombining half-molecules of two monospecific antibodies. This approach prevents chain mispairing while preserving Fc effector functions and pharmacokinetics. Several Duobody-based candidates are under clinical development for solid tumors and hematological malignancies. [10] Meanwhile, DVD-Ig antibodies consist of tandemly arranged variable domains (VH-VL) within each Fab arm, enabling simultaneous binding to two distinct epitopes. This bivalent configuration either enhances overall binding affinity or blocks two independent pathways synergistically. DVD-Ig molecules have shown promise in inflammatory and autoimmune diseases, and their potential is being explored in dual-targeted cancer immunotherapy [17]. Overall, IgG-like BsAbs have longer half-life that offers leverage effector functions. However, their large size over 150 kDa can hinder penetration into the dense PDAC tissues [18].

#### 2.1.2. Non-IgG-like BsAbs

Non-IgG-like BsAbs lack the Fc domain and are generally constructed by linking two single-chain variable fragments (scFvs). This format offers advantages such as smaller molecular size, improved tissue penetration, and flexible spatial arrangement between target-binding domains [19]. A representative non-IgG-like platform is the bispecific T-cell engager (BiTE), composed of two scFvs targeting CD3 and a tumor antigen. It redirects T cells to tumor cells to form immune synapses and induce MHC-independent cytotoxicity. Blinatumomab (CD19×CD3), the first BiTE-based therapeutic approved for clinical use, has demonstrated efficacy in treating acute lymphoblastic leukemia (ALL) by redirecting T cells for tumor cell lysis [18]. Despite its potent T-cell activation and rapid onset of action, the lack of an Fc domain results in a short half-life, necessitating continuous infusion [20]. The Dual Affinity Re-Targeting (DART) platform represents an evolution of BiTEs with enhanced structural stability by cross-linking scFvs and stabilizing the configuration through disulfide bonds. This architecture improves binding affinity and selectivity while optimizing interchain alignment for efficient T-cell redirection. Flotetuzumab (CD123×CD3), a DART-based BsAb, targets CD123-positive tumor cells in relapsed or high-risk acute myeloid leukemia (AML) and has shown promising responses in early-phase clinical trials [18,21]. In addition, TandAb is a symmetric BsAbs in which two diabody structures are linked in tandem, resulting in four antigen-binding sites. This architecture enhances binding avidity and provides more stable immune cell redirection, while exhibiting a longer serum half-life compared to conventional BiTE molecules. TandAbs are utilized in tumor-cell-killing mechanisms by engaging either CD3 or NK cell receptors, and several candidates have already advanced into clinical trials [22,23]. Bi-nanobody is an ultra-small BsAbs format constructed by linking two or more camelid-derived single-domain antibodies (nanobodies). Due to its small molecular size, non-IgG-like BsAbs typically offers excellent tumor tissue penetration, low immunogenicity, and rapid tissue diffusion, making it suitable for diverse applications such as imaging diagnostics, drug delivery, and immune modulation. More recently, bispecific nanobody strategies that recognize specific antigen pairs within the TME have been explored to overcome immune suppression [24,25].

### 2.2. Functions of BsAbs

#### 2.2.1. T-Cell Engagement

T cell engagement is the most representative function of BsAbs. By binding a T-cell (usually via CD3 on T cells) with one arm and a tumor-associated antigen with the other. This in-trans cell bridging recruits T cells to tumor cells, triggering T-cell-mediated killing of the tumor [18]. For example, blinatumomab (CD19×CD3), the first approved BiTE, is a tandem scFv fragment that can efficiently connect T cells to B-leukemia cells. It lacks an Fc (no effector function), but its small size enables agile T-cell redirection albeit with the need for continuous infusion [26]. Later, Newer T-cell engagers use IgG-like formats for better pharmacokinetics—e.g., epcoritamab (CD20×CD3) and glofitamab (CD20×CD3) are full-length IgG-like bi-specifics approved in 2022–2023. These IgG-like engagers often utilize an IgG4 or Fc-mutated backbone to minimize FcγR interactions, preventing unwanted killing of T cells (since an active IgG1 Fc could opsonize the T cell when the BsAb brings T cells and tumor together) [27]. For example, epcoritamab was engineered using Genmab’s DuoBody platform (IgG4-based), while glofitamab was engineered using Roche’s CrossMab technology. Both were created to form stable asymmetric IgG BsAbs that bridge CD3 on T cells to CD20 on B cells.

#### 2.2.2. Dual Immune Checkpoint Inhibition

Dual immune checkpoint inhibitory BsAbs are engineered to block two inhibitory receptors or ligands in tandem, with the goal of overcoming checkpoint redundancy and adaptive resistance that limit single-agent PD-1/PD-L1 or CTLA-4 therapies. Conceptually, dual blockade can operate in-cis on the same effector cell or in parallel across complementary inhibitory axes in TME, thereby amplifying T-cell activation and anti-tumor effector functions beyond monotherapy or simple antibody combinations. Recently, Bielski et al. report that anti-VISTA/anti-PD-L1 BsAbs for mitigating acquired resistance [28]. They constructed anti-VISTA/anti-PD-L1 BsAbs were constructed in three formats, symmetric IgG-HC-scFv, asymmetric Fab-scFv-Fc, and a 2×scFv design, and benchmarked against each parental monoclonal and their combination across endometrial (RL95-2), pancreatic (PANC-1), and breast (BT-20) models. In the preclinical study, Fc-based BsAbs produced significantly higher tumor-cell lysis and greater secretion of pro-inflammatory mediators (e.g., Interferon-gamma (IFN-γ), Tumor necrosis factor-alpha (TNFα), Granzyme B) than either monotherapy or the antibody combination, with the symmetric IgG-HC-scFv format emerging as most promising [28]. Collectively, these results indicate that dual immune-checkpoint blockade integrated within a single bispecific scaffold can surpass the activity of separate agents while mechanistically aligning with the need to counter checkpoint redundancy in solid tumors such as pancreatic cancer.

#### 2.2.3. Dual Tumor Antigen Targeting

BsAbs designed for dual tumor antigen targeting represent an innovative approach to improve therapeutic specificity while minimizing off-target toxicities. Unlike monospecific antibodies, these molecules are engineered to simultaneously bind two distinct antigens that are co-expressed on the same tumor cell. This strategy enables in-cis recognition, where binding requires the concurrent presence of both targets, thereby reducing nonspecific interactions with normal tissues [29,30]. In addition, dual antigen binding can induce avidity effects, whereby the combined affinity enhances tumor selectivity compared to single-target engagement [31]. Mechanistically, dual targeting BsAbs can exert in-cis antagonism or neutralization of parallel oncogenic pathways, thus reducing the likelihood of resistance arising from compensatory signaling [30,32]. This principle has been exemplified in clinical settings: amivantamab, which targets EGFR and MET, demonstrates the potential of dual receptor blockade in overcoming resistance mechanisms in lung cancer, while faricimab, targeting VEGF-A and Ang-2, illustrates the utility of dual ligand neutralization in modulating angiogenic pathways [33,34]. Collectively, these cases underscore the therapeutic value of dual tumor antigen targeting BsAbs as next-generation modalities in oncology and beyond.

#### 2.2.4. Receptor Clustering

Receptor clustering represents a distinct mechanism of BsAbs, whereby a single molecule simultaneously binds two receptors and enforces their spatial proximity. This process functions as a synthetic agonist, promoting receptor cross-linking and subsequent signaling cascades [31]. Typically, clustering occurs in-cis, with both receptors located on the same cell surface, and can be exploited to selectively activate immune co-stimulatory receptors or to trigger apoptotic pathways in tumor cells. A major challenge of this approach is the risk of systemic activation, which may result in severe immune-related toxicities; thus, conditional activation is essential for clinical translation [31,35]. To address this limitation, BsAbs have been engineered as conditional agonists, inducing receptor clustering only under tumor-associated conditions. A prototypical example is acasunlimab (GEN1046/BNT311), an IgG-like DuoBody BsAbs that targets PD-L1 on tumor cells and 4-1BB on T cells. This design ensures that 4-1BB clustering and activation occur only in the presence of PD-L1, thereby coupling checkpoint blockade with localized T-cell co-stimulation in the TME [35]. Its Fc domain is further silenced to prevent non-specific cross-linking and Fc-mediated effector functions, enhancing safety. Similarly, tetravalent PD-L1×4-1BB constructs such as ATG-101 have demonstrated broad therapeutic windows in preclinical studies, eliciting strong anti-tumor immunity while avoiding systemic cytokine release and hepatotoxicity [36,37]. Additional strategies include CD28×tumor antigen BsAbs, which deliver co-stimulatory signals exclusively upon binding to tumor cells, thereby improving both selectivity and therapeutic safety [38]. Collectively, receptor clustering BsAbs illustrate how rational antibody engineering can integrate checkpoint inhibition and conditional co-stimulation into a single molecule. Among the various designs, Fc-silenced IgG-like formats remain the preferred architecture, balancing conditional agonist activity with extended serum half-life and favorable pharmacokinetic properties.

#### 2.2.5. Cytokine or Cofactor Mimicking

Cytokine- or cofactor-mimicking BsAbs are designed to replicate the functional activity of natural cytokines or cofactors. These molecules achieve their effect by simultaneously binding two distinct targets—either proteins or receptor subunits—and thereby enforcing an interaction that would normally require a soluble mediator. Through this mechanism, BsAbs can activate downstream biochemical pathways that are otherwise impaired in disease states [10]. One clinically validated example is emicizumab (Hemlibra^®^), an IgG4 BsAb that simultaneously binds activated factor IX (FIXa) and factor X (FX). By mimicking the cofactor activity of factor VIII, emicizumab restores tenase complex formation in patients with hemophilia A, thereby normalizing thrombin generation and blood clotting [39,40]. Its Fc-silenced IgG4 backbone reduces unwanted immune effector activity, and its extended half-life supports convenient subcutaneous dosing. Importantly, emicizumab represents the first FDA-approved cofactor-mimetic BsAb, highlighting the therapeutic potential of this strategy [39]. Beyond coagulation, cytokine-mimetic BsAbs are being investigated in oncology. For example, IL-15 superagonist constructs have been developed to bridge IL-15 with IL-15Rβγ selectively within the TME, enhancing local T and NK cell activity while limiting systemic toxicity [41]. Other approaches include BsAbs engineered to induce receptor dimerization in the absence of a natural ligand, effectively substituting for cytokine-mediated signaling [10]. Taken together, cytokine- and cofactor-mimicking BsAbs demonstrate how rational antibody engineering can recreate essential biological functions without reliance on native cofactors. Owing to the need for long-term pharmacological activity, most of these molecules adopt IgG-like architectures, which provide stability, extended serum half-life, and suitability for chronic therapeutic use [10].

### 2.3. Examples of BsAbs in PDAC Treatments

Currently, a few BsAbs are in clinical trials as PDAC treatments. Table 1 summarizes these agents, their targets and mechanisms, and their current development status, along with key findings from studies to date.

**Figure 2:**
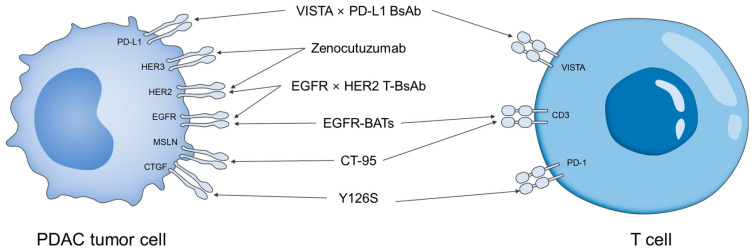
Schematic illustration of BsAbs in PDAC and their target ligands.

Based on recent clinical findings, BsAbs in PDAC have shown several notable achievements. As shown in Figure 2, four bispecific antibodies (BsAbs) have been developed to target multiple antigens, and two BsAbs have been developed to engage T cells. For example, zenocutuzumab achieved clinical success in a subset of patients harboring NRG1 fusions, representing the first FDA approval of a BsAb for pancreatic cancer [51]. This milestone demonstrates that dual-targeting antibodies can provide meaningful benefits in carefully defined patient populations. Furthermore, preclinical studies have validated T-cell redirection strategies, showing that BsAbs can recruit cytotoxic lymphocytes into TME and directly induce tumor cell killing [52]. These findings suggest that BsAbs are not only able to overcome immune evasion but may also expand into broader applications, including their potential role in companion diagnostics and advanced drug delivery systems. In addition, BsAbs are being actively explored in combination with conventional chemotherapies and immune checkpoint inhibitors, aiming to enhance therapeutic efficacy and overcome resistance to monotherapies [53].

Nevertheless, important limitations must also be acknowledged. The highly immunosuppressive TME, characterized by dense stroma, hypoperfusion, and suppressive immune cell populations, continues to hinder immune activation and drug penetration [52]. Antigen heterogeneity further complicates therapeutic applicability, as expression patterns vary across tissues and patients, limiting the breadth of treatment. Safety concerns remain critical, including cytokine release syndrome, immune-related adverse events, and the burden of intravenous administration [52]. Clinical data are still insufficient, with most findings derived from preclinical or early-phase studies, and overall survival outcomes remain uncertain [52]. From a developmental perspective, the structural complexity of BsAbs leads to high manufacturing costs, challenging scalability, and accessibility [53]. Finally, resistance mechanisms, such as downregulation of target antigen expression or secretion of immunosuppressive cytokines, may compromise the long-term durability of therapeutic responses [53].

Meanwhile, neither Abs nor ADCs have yet received clinical approval for PDAC treatment (Table 2) [54]. It is noteworthy that Abs and ADCs are being developed to target PDAC-specific TAAs, such as CLDN18.2, MUC1, uPAR, EphA2, CEACAM5, Trop2. Currently, the majority of BsAbs are used to treat PDAC target common antigens found in cancer, such as HERs, EGFR, and PD-L1, rather than being specific to PDAC [30,55]. This is because common cancer antigens are targets for which the clinical safety and efficacy of Abs or ADCs has already been extensively validated [56]. These antigens have the advantage of a relatively predictable toxicity profile, enabling a rapid transition from preclinical to clinical trials. Furthermore, targets that are widely expressed across multiple solid tumours (e.g., breast, lung, colorectal, and pancreatic cancers) are preferred due to their broad applicability and significant commercial and clinical potential [30,56]. This is because the inherently complex structure of BsAbs, compared to standard antibodies, results in higher development costs. Furthermore, the target for BsAbs must be selected with due consideration of their unique mechanism of action [57].

**Table 2:** Abs and ADCs in clinical trials for PDAC.

Types	Names	Targets	Disease	Company	Development Stage	NCT.	Ref.
Abs	HuMab-5B1 (MVT-5873)	CA19-9	Pancreatic ductal adenocarcinoma, biliary cancers	Sorrento/MabVax Therapeutics	Phase 1	NCT02672917	[58,59]
	**ensituximab** (NPC-1C/NEO-102)	MUC5AC-related tumor antigen	Refractory colorectal and pancreatic cancer	Precision Biologics/Neogenix	Phase 1/2	NCT01834235	[60,60]
ADCs	IBI343	CLDN18.2	Pancreatic ductal adenocarcinoma (PDAC), biliary tract cancer (BTC)	Innovent Biologics	Phase 1	NCT05458219	[61,62]
	EBC-129	CEACAM5/CEACAM6	Pancreatic ductal adenocarcinoma (PDAC)	Experimental Drug Development Centre	Phase 1	NCT05701527	[63,64]
	OMTX705	FAP	Advanced pancreatic adenocarcinoma		Phase 1b	NCT05547321	[65,66]
	uPAR-ADC	uPAR	Pancreatic ductal adenocarcinoma (PDAC)	Oncomatryx/TFS HealthScience	Preclinical	no NCT	[67]
	GPC-1 ADC	GPC-1	GPC1-positive pancreatic cancer	University of Copenhagen/Genmab	Preclinical	no NCT	[68]
	TR1801-ADC	c-MET	KRAS-mutated PDAC/resistant tumors	Kyowa Kirin/Osaka University	Preclinical	no NCT	[69]

## 3. Bispecific ADCs and Their Therapeutic Potential in PDAC

### 3.1. Definition of BsADCs

BsADCs are structures comprising an antibody, a linker and a payload, which integrates the capacity of antibody–drug conjugates (ADCs) to deliver cytotoxic drugs with the dual-targeting capability of BsAbs. With the ability of BsAbs, BsADCs can recognize two antigens or epitopes simultaneously, enhancing tumor specificity and increasing intracellular uptake efficiency. This dual-targeting mechanism enables precise recognition of malignant cells while reducing nonspecific binding to normal tissues, thereby minimizing off-target toxicity [11,70]. Structurally, BsADCs have the potential to simultaneously inhibit multiple cancer-related signaling pathways by simultaneously targeting cell surface ligands and intracellular metabolic pathways. Therefore, BsADCs have emerged as a next-generation therapeutic platform by mitigating tumor heterogeneity and drug resistance, which have challenged conventional chemotherapies and mAb therapies [70,71].

#### 3.1.1. Linkers of BsADCs

In BsADCs, the linkers serve as a structural cornerstone that connects the antibody to the cytotoxic payload, critically influencing pharmacological behavior, in vivo stability, and overall therapeutic efficacy [11,71]. Linkers are broadly classified into cleavable and non-cleavable types, and this classification fundamentally influences drug release mechanisms and tissue specificity [11].

Cleavable linkers are designed to selectively dissociate in response to the unique biochemical environment within tumor cells. Prominent triggers include acidic pH, high concentrations of glutathione (GSH), and lysosomal enzymes such as cathepsin B, which are typically more pronounced in tumor cells than in normal tissues. Leveraging these conditions, cleavable linkers enable payload release predominantly within tumor cells, thereby minimizing off-target toxicity while maximizing therapeutic specificity and efficacy [70,72].

Non-cleavable linkers possess high chemical stability, resisting degradation under both extracellular and intracellular physicochemical conditions. In BsADCs containing non-cleavable linkers, the conjugate is internalized into the cell, where lysosomal degradation of the antibody releases the payload. At this stage, the payload remains covalently bound to amino acid residues of the antibody, typically via thioether bonds [11]. This structural feature significantly enhances systemic stability and effectively prevents premature drug leakage. However, since the released payload exists in a chemically modified form, its intracellular cytotoxic activity may sometimes be reduced [11.

Therefore, linkers selection in BsADCs design requires comprehensive consideration of TME, the internalization capacity of the target antigen, the structural sensitivity of the payload, and its pharmacodynamic properties. These factors collectively act as pivotal variables that determine the balance between efficacy and stability in the therapeutic system [11,72].

#### 3.1.2. Payload of BsADCs

The payload of BsADCs must exhibit sufficient potency to induce apoptosis within target cells while also maintaining chemical stability in conjugation with the antibody and preserving both activity and structural integrity in the linker-bound state [73,74]. The most widely employed payload classes are microtubule inhibitors (monomethyl auristatin E, MMAE; monomethyl auristatin F, MMAF), DNA-damaging agents (calicheamicin; pyrrolobenzodiazepine, PBD), and topoisomerase I inhibitors (SN-38; deruxtecan, DXd). The features of each payload class are shown in Table 3.

These agents exert strong cytotoxic effects even at extremely low concentrations, and notably, topoisomerase I inhibitors have recently emerged as pivotal payloads in the development of diverse BsADCs [11,92]. In clinical applications, trastuzumab deruxtecan (DXd) and sacituzumab govitecan (SN-38) have demonstrated improvements in progression-free and overall survival in HER2-low breast cancer and metastatic triple-negative breast cancer, respectively, thereby validating the clinical utility of the topoisomerase I inhibitor class [93,94]. Payload selection should not be based solely on cytotoxic potency but must also incorporate considerations of antibody internalization capacity, linker release mechanisms, and physicochemical properties, alongside the optimization of pharmacokinetics and pharmacodynamics (PK/PD) [73,74].

### 3.2. Rationales of BsADCs in PDAC Treatments

BsADCs are composed of an antibody scaffold bearing two antigen-binding sites conjugated via a linker to a cytotoxic payload, thereby combining the targeting precision of antibodies (Abs, BsAbs) with the drug delivery capability of ADCs to enhance therapeutic efficacy [74,95]. Additionally, BsADCs offer significant advantages, as a therapeutic strategy to block complex oncogenic signaling pathways. While a single antibody can inhibit only one pathway, BsADCs are capable of simultaneously suppressing parallel or compensatory pathways, thereby enhancing both the intensity and durability of antitumor effects. This dual-pathway inhibition provides a critical strategic benefit for tumor therapies that rely on multi-pathway targeting [96].

Compared to Abs, BsADCs are designed to recognize combinations of antigens that are co-expressed on the surface of tumor cells. It can remarkably reduce the likelihood of nonspecific binding to normal cells while markedly improving tumor specificity [96,97]. In particular, by simultaneously attacking cell populations expressing different antigens, it can effectively reduce treatment evasion caused by antigen heterogeneity and antigen loss, which are commonly observed in PDAC. As a result, BsADCs can minimize off-target toxicity and enable more precise tumor targeting [11]. Furthermore, in cases where antigen expression is downregulated or lost due to mutations, conventional antibody-based therapeutics often fail to sustain drug activity. By contrast, BsAbs can effectively control compensatory signaling activation that is difficult to inhibit with single antibodies by simultaneously blocking different signaling pathways (e.g., EGFR–HER2, MET–HER3) [54]. In PDAC, downstream signaling pathways like KRAS mutations remain active after antibody blockade, readily leading to resistance. However, the dual-target approach simultaneously suppresses parallel upstream pathways, slowing the development of resistance and enhancing the durability of therapeutic response [98]. This dual-target format also confers advantages in overcoming therapeutic resistance [65].

Meanwhile, BsADCs possess a distinct cytotoxic mechanism by releasing their payloads intracellularly through receptor-mediated internalization and lysosomal linker cleavage. For cytotoxic payloads to exert their therapeutic effects, efficient and stable intracellular uptake is essential, and the structural design of BsADCs effectively enable this process. Dual-targeting further enhances this function by pairing antigens with slow or negligible internalization capacity to those with rapid internalization, thereby promoting uptake and trafficking of the entire complex. Consequently, BsADCs can expand their therapeutic reach to target populations that are largely inaccessible to conventional ADCs or Abs [74,95,99].

Additionally, BsADCs can target the heterogenous TME that is intractable for conventional ADCs. Intertumoral heterogeneity in antigen expression is one of the key factors limiting the therapeutic efficacy of single-antibody-based treatments. By simultaneously recognizing distinct antigens, BsADCs enable comprehensive targeting of diverse tumor cell subtypes, thereby contributing to consistent drug efficacy even within complex TME [30]. When antigen heterogeneity restricts the effects of Abs or BsAbs, BsADCs can broaden coverage across multiple subtypes. When paired with payload–linker combinations capable of exerting bystander effects, BsADCs can indirectly induce cytotoxicity in antigen-negative cells [75,100]. Moreover, for targets that are poorly internalized, BsADCs can incorporate an internalizing antigen as a co-target, thereby securing alternative pathways for drug uptake. This strategy is regarded as a valid approach to expanding therapeutic options for low-activity targets, which are often unaddressed by conventional Abs or BsAbs [101].

Furthermore, from a pharmacokinetic perspective, bispecific antibodies (BsAbs) can enhance tissue penetration and speed of action by employing small formats, such as BiTE and DART. Certain structures can also optimise drug distribution and accumulation within the body by modulating half-life [31,73]. In PDAC, which has a hypovascular structure, such modifications to half-life and binding affinity can improve drug accessibility within the tumour [102]. Compared to monoclonal antibodies or ADCs, which generally exhibit consistent distribution patterns, BsAbs/BsADCs have the potential advantage of leveraging structural diversity to finely tune tissue penetration, binding strength and range of action [73].

In summary, BsAbs and BsADCs are being evaluated as flexible design platforms that can simultaneously overcome complex antigen expression heterogeneity and resistance mechanisms in PDAC, thus surpassing single-antibody-based therapies. The dual-target strategy, recognizing two targets in parallel or complementarily, is gaining attention as a key approach to overcoming the limitations of existing therapies, such as antigen loss, resistance and toxicity, and maximising therapeutic efficacy in PDAC.

### 3.3. Recent Progress of BsADCs in Anticancer Therapies

To date, no BsADCs for the treatment of PDAC are currently in clinical trials. Meanwhile, several BsADCs have been used in anti-cancer therapies, as shown in Table 4. Notably, a few BsADCs have reached a stage of clinical advancement, such as AZD9592 (EGFR×c-MET), M1231 (MUC1×EGFR), and BL-B01D1 (EGFR×HER3) [103,104,105]. AZD9592 (tilatamig samrotecan) is an IgG1 BsAbs, targeting EGFR and c-MET, conjugated through a cleavable peptide linker to a CPT (camptothecin)-class topoisomerase I inhibitor (AZ14170132, samrotecan). Preclinical studies showed that AZD9592 correlated with target expression and induced DNA double-strand break markers such as γH2AX in EGFR–MET co-expressing solid tumors [106,107,108]. Currently, a multinational first-in-human (FIH) phase I study (EGRET, NCT05647122) is evaluating both monotherapy and combinations with dose-escalation and expansion cohorts. According to recent studies, the clinical practice remains in the stage of assessing safety, pharmacokinetics/pharmacodynamics (PK/PD), and preliminary antitumor activity, with efficacy outcomes such as objective response rate (ORR) not having been reported so far [103,107].

M1231 consists of an MUC1×EGFR BsAb conjugated via a Val-Cit-PABA cathepsin-cleavable linker (drug–antibody ratio [109] ≈ 4) to SC209, a hemiasterlin-derived microtubule inhibitor. In preclinical patient-derived xenograft (PDX) models of non-small-cell lung cancer (NSCLC) and esophageal squamous cell carcinoma (ESCC), M1231 exhibited potent antitumor activity including complete remission (CR). Mechanistically, M1231 showed enhanced co-internalization and lysosomal trafficking compared to monospecific bivalent antibodies [110]. A phase I clinical trial (NCT04695847) is currently in the dose-escalation and expansion phase, focusing mainly on NSCLC and ESCC. At this stage, the available data emphasize tolerability, recommended dose exploration (RDE/MTD), and preliminary efficacy signals, however, definitive clinical outcomes have not yet been established [104].

Among them, BL-B01D1 represents the most advanced clinical program. This EGFR×HER3 BsAb is conjugated at a high drug–antibody ratio (~8) via a cathepsin B–cleavable linker to Ed-04, a topoisomerase I inhibitor. In a phase I (a/b) analysis, an ORR of 34% (60/174; 95% CI 27–42) was reported, thereby establishing a recommended phase II dose (RP2D) of 2.5 mg/kg administered on days 1 and 8 of a 3-week cycle. In terms of safety, treatment-related grade ≥3 adverse events were observed in 71% of patients including neutropenia (47%), anemia (39%), leukopenia (39%), and thrombocytopenia (32%). In addition, treatment-related mortality occurred in 2% of patients, and a single case of interstitial lung disease (ILD) was reported. [105]. Subsequently, the platform has been extended to additional indications, with efficacy signals observed in an ESCC phase Ib cohort. Furthermore, multiple global expansion studies are being conducted, including combination regimens in renal cell carcinoma with axitinib×pembrolizumab (NCT06962787) [9,111,112].

Taken together, these three agents represent the expanding clinical scope of BsADCs utilizing topoisomerase I inhibitor- and microtubule inhibitor-based payloads. Notably, BL-B01D1 is currently the most advanced program, with validated dosing, scheduling, and objective response data [105].

**Table 4:** BsADCs in clinical trials for anticancer therapies.

Development Stage	BsADCs (Targets)	Payload	Disease	Company	NCT.	Ref.
Preclinical stage	BVX001 (CD7×CD33)	Monomethyl auristatin F (MMAF)	Acute Myeloid Leukemia (CD7+/CD33+)	BiVictriX Therapeutics	×	[113,114]
	VBC103 (Trop2×Nectin4)	Topoisomerase I inhibitor	Urothelial Carcinoma (metastatic), Triple-Negative Breast Cancer (+ Othesrs)	VelaVigo, Avenzo Therapeutics	×	[115]
Phase1	AZD9592 (EGFR×c-MET)	Topoisomerase I inhibitor (“samrotocan”)	Advanced solid tumors (EGFR×c-MET co-expressing; NSCLC focus)	AstraZeneca	NCT05647122	[103,116]
	ZW-49 (HER2 (ECD2×ECD4))	Auristatin (microtubule inhibitor)	HER2-expressing solid tumors (breast, gastric, etc.)	Zymeworks	NCT03821233	[117,118]
	JSKN0016 (HER3×TROP2)	Topoisomerase I inhibitor	Advanced solid tumors (basket, incl. lung, breast)	Alphamab Oncology	NCT06868732	[119,120]
	M1231 (MUC1×EGFR)	SC209 (microtubule disruptor)	Advanced solid tumors (dose escalation); NSCLC & ESCC (expansion cohorts)	EMD Serono, Sutro Biopharma	NCT04695847	[104,110]
	DM001 (EGFR×TROP2)	Monomethyl auristatin E (MMAE)	Advanced solid tumors (breast, EGFR-mut/wt NSCLC, gastric, esophageal, colorectal)	XADCera	NCT06475937	[121,122]
	DM005 (EGFR×c-MET)	Topoisomerase I inhibitor	Advanced solid tumors (NSCLC, H&N, GI, etc.)	Doma Biopharm	NCT06515990	[123,124,125]
	BL-B01D1 (EGFR×HER3)	Ed-04 (topoisomerase I inhibitor)	Multiple solid tumors (NSCLC, breast, etc.)	Sichuan Baili, SystImmune	NCT05983432	[9,105,126]
	BL-B01D1 (EGFR×HER3)	Ed-04 (topoisomerase I inhibitor)	Advanced solid tumors (Phase 1a dose escalation); Expansion in ESCC and other GI cancers	Sichuan Baili, SystImmune	NCT05262491	[9,105,127]
	BL-B16D1 (EGFR×HER3)	Monomethyl auristatin E (MMAE)	Advanced solid tumors (all-comers)	Sichuan Baili, SystImmune	NCT06475131	[128]
	BL-B16D1 (EGFR×HER3)	Monomethyl auristatin E (MMAE)	Head & Neck Squamous Cell Carcinoma (+ others)	Sichuan Baili, SystImmune	NCT06469008	[129]
	BL-B16D1 (EGFR×HER3)	Monomethyl auristatin E (MMAE)	HER2-negative Breast Cancer (+ others)	Sichuan Baili, SystImmune	NCT06493864	[130]
	IBI-3001 (B7-H3×EGFR)	Undisclosed	Advanced solid tumors (B7-H3 & EGFR co-expressing)	Innovent Biologics	NCT06349408	[131,132,133]
Phase1/2	REGN5093-M114 (MET biparatopic)	Maytansine derivative (M24)	Non-Small-cell Lung Cancer (NSCLC; MET-overexpressing; advanced)	Regeneron Pharmaceuticals	NCT04982224	[134,135]
	MEDI4276 (HER2 (ECD2×ECD4)	AZ13599185 (microtubule inhibitor)	Breast & Gastric Cancer (HER2-overexpressing; advanced)	MedImmune, AstraZeneca	NCT02576548	[136,137]
	GEN1286 (EGFR×c-MET)	Topoisomerase I inhibitor	Advanced solid tumors (ovarian, NSCLC, gastric, etc.)	Genmab	NCT06685068	[138,139]
	VBC101- (EGFR×c-Met)	Monomethyl auristatin E (MMAE)	Solid tumors co-expressing EGFR & MET	VelaVigo Bio	NCT07136779	[140,141,142]
Phase2	BL-B01D1 (EGFR×HER3)	Ed-04 (topoisomerase I inhibitor)	Renal Cell Carcinoma (locally advanced or metastatic)	Sichuan Baili, Bristol Myers Squib	NCT06962787	[9,105,143]
Phase3	TQB2102 (HER2 (ECD2×ECD4))	Topoisomerase I inhibitor	Breast Cancer (HER2+, early stage, neoadjuvant)	Chia Tai Tianqing	NCT07043725	[144,145]
	JSKN003 (HER2 biparatopic)	Topoisomerase I inhibitor	Ovarian Cancer (HER2-expressing, platinum-resistant)	lphamab Oncology	NCT06751485	[146]
	JSKN003 (HER2 biparatopic)	Topoisomerase I inhibitor	HER2-positive Breast Cancer (advanced, post-T-DM1)	lphamab Oncology	NCT06846437	[147,148]
	BL-B01D1 (EGFR×HER3)	Ed-04 (topoisomerase I inhibitor)	Triple-Negative Breast Cancer (metastatic)	Sichuan Baili, Bristol Myers Squib	NCT06382142	[9,105,149]
	BL-B01D1 (EGFR×HER3)	Ed-04 (topoisomerase I inhibitor)	HR+/HER2– Metastatic Breast Cancer	Sichuan Baili, Bristol Myers Squib	NCT06343948	[9,105,150]
	BL-B01D1 (EGFR×HER3)	Ed-04 (topoisomerase I inhibitor)	Esophageal squamous cell carcinoma (2L, post-IO)	Sichuan Baili, Bristol Myers Squib	NCT06304974	[9,105,151]

### 3.4. Recent Progress of BsADCs for PDAC Treatments

**Figure 3:**
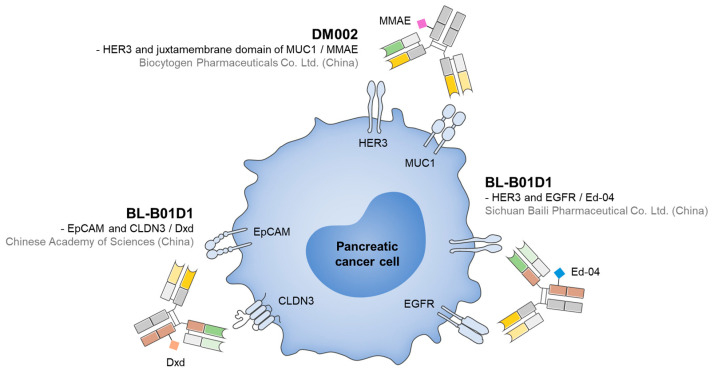
Representative BsADCs for PDAC treatments, along with their target ligands and payloads.

To date, the development of BsADCs for PDAC treatment has remained in the early stages, but several drug candidates have demonstrated potential in preclinical studies (Figure 3). For example, EpCAM×CLDN3 BsADC, engineered using CrossMab and Knobs-into-Holes technologies and conjugated with DXd via a GGFG-based cleavable linker, exhibited potent cytotoxicity with an IC_50_ of 0.72 μg/mL in EpCAM^+^/CLDN3^+^ cell lines and induced significant tumor regression in xenograft models while maintaining favorable safety and pharmacokinetic stability [109]. Notably, it shows low activity in EpCAM-high/CLDN3-low models, suggesting the rationale for AND-gating-based specificity providing reduced off-target toxicity. Another example is HER3×MUC1 BsADC (DM002 series). It was developed in two formats, DM002-vcMMAE and DM002-BLD1102, both of which enhanced co-internalization and intracellular drug delivery, resulting in excellent antitumor efficacy in cell-line-derived xenograft and patient-derived xenograft tumor models. DM002-vcMMAE consistently outperformed its parental ADC, while DM002-BLD1102 maintained or exceeded efficacy in vcMMAE-resistant models, suggesting that payload class switching may overcome acquired resistance [152,153]. In the meantime, mesothelin-targeting ADC (DMOT4039A) demonstrated partial responses in a subset of pancreatic cancer patients in a phase I trial, with a manageable safety profile, thereby validating mesothelin as a promising target in pancreatic cancer [154]. Therefore, these findings highlight that BsADCs have achieved clear preclinical and early clinical successes, providing a rationale for their further exploration in PDAC.

Among the ongoing programs, EGFR×HER3 BsADC (BL-B01D1) represents the most clinically advanced candidate. In a phase I trial including 174 evaluable patients with advanced solid tumors, BL-B01D1 achieved an ORR of 34% and established an RP2D of 2.5 mg/kg on Days 1 and 8 of a 3-week cycle. Safety outcomes were generally manageable, and some patients experienced durable responses [105]. These data support the translational feasibility of BsADCs in overcoming resistance and broadening the therapeutic utility of ADC platforms in PDAC [36].

## 4. Discussion and Future Perspectives

Through the efforts of many researchers, the potential for treating PDAC using BsAbs and BsADCs has been validated, and reports demonstrating its clinical utility are gradually increasing [54,56]. Nevertheless, the unique tumor microenvironment and biological characteristics of PDAC have hindered the practical application of BsADCs thus far. For example, the dense ECM and elevated interstitial pressure in PDAC markedly reduce the diffusion and tissue penetration of large antibody molecules and their payloads, resulting in poor intratumoral drug distribution [102]. Moreover, the poor vascular perfusion and hypovascularity of PDAC further restrict antibody delivery compared with highly vascularized cancers such as breast or lung tumors [155]. In addition, tumour antigens are heterogeneous, and inconsistent co-expression of dual targets limits the reliability of dual-targeting of BsADCs (AND strategy) and conditional or sequential targeting (OR strategies) [54]. Moreover, frequent targets, such as TROP2, MUC1, and mesothelin, are also expressed at low levels in normal tissues, therefore raising concerns of off-target toxicities [156,157]. Furthermore, PDAC exhibits a “cold tumor” immune phenotype characterized by low T-cell infiltration and a predominance of immunosuppressive cell populations such as regulatory T cells, tumor-associated macrophages (TAMs), and myeloid-derived suppressor cells (MDSCs), which collectively limit the efficacy of T-cell-engaging BsAbs [158]. Finally, reduced tumor perfusion decreases overall antibody delivery efficiency, while nonspecific interstitial binding within the fibrotic stroma prolongs tissue residence time, leading to payload leakage and increased off-tumor toxicity [159]. Collectively, these factors complicate pharmacokinetic (PK) predictability and narrow the effective dose window. Meanwhile, payload-related toxicities, including ILD observed with DXd-based agents, further constrain the therapeutic window [156]. Resistance mechanisms such as antigen downregulation, drug efflux, and enhanced DNA repair also limit durable responses [160,161,162]. In this regard, despite their potent payloads and encouraging preclinical activity, ADCs have so far produced disappointing results in clinical trials for PDAC. Taken together, while BsADCs have shown significant promise in both preclinical and early clinical contexts, overcoming biological and technical hurdles remains essential to realize their therapeutic potential in PDAC [54].

First, a deep understanding of the in vivo behavior of engineered nanomaterials is required [163]. Unlike biogenic mAbs, BsAbs and BsADCs are heterogeneous nanomaterials. Therefore, their intrinsic behaviors as antibodies, such as binding to Fc receptors or Fc recycling, may differ significantly from those of mAbs. Therefore, in-depth studies on the distribution behavior of BsAbs and BsADCs under various in vivo conditions, as well as their in vivo distribution in PDAC models, appear necessary [102,164].

Second, strategies to overcome the demoplastic and immunosuppressive environment of pancreatic cancer must be considered. Beyond T-cell-engaging strategies, which have shown clinically successful outcomes, introducing additional strategies to enhance T cell function and control the function of cancer-associated fibroblasts that generate tolerogenic signals could provide a novel approach to maximize the synergistic effects of the cytotoxic payload and immunosurveillance effects of BsADCs [159].

Third, consideration is needed for strategies to overcome the low perfusion and high interstitial pressure in pancreatic cancer. For PDAC, where particle extravasation into tissue is inefficient, binding to claudins or specific binding to albumin could increase accumulation rates in pancreatic tissue. This, coupled with extended half-lives, could provide BsAbs and BsADCs with more opportunities to bind their targets [165,166]. Another example is increased tissue penetration through fragmentation. The nanosatellite strategy, one of the penetration strategies for nanomaterials, involves components fragmenting into smaller sizes specifically within the target tissue, enabling effective penetration into the tissue interior [167]. Similarly, if the linkers connecting the fragments composing BsAbs are designed to be selectively cleavable within the TME, large structures like IgG-like BsAbs or TandAbs could be expected to dissociate into smaller bispecific fragments within the microenvironment, thereby enhancing penetration efficiency into solid tumors.

## Figures and Tables

**Table 1:** Examples of BsAbs in PDAC treatment.

BsAbs (Targets)	Strategy/Mechanism	Development Stage	Key Findings in PDAC	Ref.
zenocutuzumab (HER2×HER3)	Dual tumor receptor blockade (in NRG1 fusion-driven tumors)	Phase II–Approved (2024)	First FDA-approved BsAb for PDAC with NRG1 fusions; blocks HER2/HER3 signaling. In NRG1+ PDAC, achieved 40% ORR with durable responses up to 16.6 months.	[42,43,44]
VISTA×PD-L1 BsAb	Dual immune checkpoint inhibitor	Preclinical (in vitro)	Simultaneous VISTA and PD-L1 blockade. In PANC-1 cell assays, IgG-based BsAb induced higher tumor cell lysis and IFN-γ, TNFα, Granzyme B secretion than single-agent or combo therapy.	[28]
EGFR×HER2 T-BsAb	T-cell engaging bispecific (IgG-[L]-scFv format) targeting two tumor antigens	Preclinical (in vitro, xenograft)	Heterodimeric IgG-scFv linking anti-EGFR and anti-HER2 with anti-CD3 scFv to recruit T cells. Demonstrated picomolar-range cytotoxicity in vitro and potent T cell-mediated tumor regression in PDAC xenografts. Required co-expression of both targets for efficacy, suggesting improved tumor specificity.	[45]
Y126S (CTGF×PD-1 BsAb)	Stromal targeting + checkpoint blockade	Preclinical (mouse models)	IgG1/IgG2 hybrid BsAb targeting desmoplastic stroma (CTGF) and T cell checkpoint (PD-1). Remodels PDAC TME: suppresses cancer associated fibroblast activation, reduces collagen, downregulates PD-L1 on stroma, and boosts CD8+ T cell activity, leading to enhanced tumor suppression vs. monotherapies.	[46]
EGFR-BATs (CD3×EGFR bispecific-armed T cells)	Adoptive cell therapy with BsAb-armed T cells	Phase I completed; Phase Ib/II ongoing	Autologous T cells coated with anti-CD3×EGFR BsAb to redirect T cells to EGFR^+ tumor cells. Safe in Phase I (no DLTs); showed immune activation and clinical benefit (stable disease ≥6 months in some cases). Small study reported median OS 31 months in heavily pretreated PDAC, with occasional complete responses when combined with chemo.	[47,48]
CT-95 (Mesothelin×CD3 BsAb)	T-cell engaging bispecific (targets tumor antigen MSLN)	Phase I (2025, recruiting)	Fully human T-cell engager for mesothelin-expressing solid tumors (incl. PDAC). Aims to direct T cells to MSLN^+ cancer cells; first patient dosed in 2025. Initial trial results expected 2026; preclinical data suggest high avidity binding and tumor-specific T cell lysis.	[49,50]

**Table 3:** Payload candidates of BsADCs for PDAC treatments.

Payload Class	Specific Payload	Mechanism	Example ADCs in PDAC	Preclinical or Clinical Findings	Ref.
Microtubule Inhibitors	MMAE (monomethyl auristatin E)	Tubulin polymerization inhibitor (M-phase arrest)	Anti-SLC44A4 (ASG-5ME), Anti-Tissue Factor ADCs, Anti-GPC1 ADCs	Potent tumor regressions in PDAC xenografts; MMAE enabled bystander effect (killed antigen-negative cells via diffusion). XB002 (TF-MMAE ADC) in early trials including PDAC.	[54,75,76]
	DM1 (emtansine)	Microtubule destabilizer (non-cleavable linker, no bystander effect)	trastuzumab emtansine (T-DM1, HER2 ADC)	Tested in HER2 ^+^ PDAC: only 1/7 responses; limited efficacy (short PFS).	[54,77]
	DM4 (ravtansine)	Tubulin inhibitor (cleavable linker, allows for bystander effect)	anetumab ravtansine (anti-Mesothelin)	Stable disease in subset of PDAC patients; limited responses but synergy with checkpoint inhibitors + gemcitabine (100% disease control in one cohort).	[78,79,80]
DNA-Damaging Agents	Duocarmycin analogs	DNA alkylation (minor groove binding)	TAK-164 (anti-GCC, duocarmycin) and MGC018 (anti-B7-H3, duocarmycin)	TAK-164 halted early despite rationale; MGC018 in Phase I (including PDAC).	[54,81,82,83]
	PBD Dimers (tesirine, etc.)	DNA crosslinking (non-cell-cycle dependent, not effluxed)	TR1801-ADC (anti-MET, PBD), ADCT-601 (anti-AXL, PBD)	Strong tumor inhibition in PDAC xenografts, esp. combined with gemcitabine; effective even in resistant tumors. Narrow therapeutic index remains challenge.	[84,85]
	Anthracyclin analog (PNU-159682)	DNA intercalation + strand breaks (extremely potent)	SOT102 (anti-Claudin 18.2, PNU-159682)	Preclinical regressions in Claudin18.2 ^+^ tumors; Phase I/II trial ongoing (includes GI cancers like PDAC).	[86]
Topoisomerase Inhibitors	SN-38(active metabolite of irinotecan)	Topoisomerase I inhibition → DNA strand breaks	labetuzumab govitecan (anti-CEA-SN-38), sacituzumab govitecan (anti-Trop-2-SN-38)	In PDAC xenografts: significantly better than irinotecan (prolonged survival, tumor regression). In clinical PDAC: modest benefit (stable disease in ~40%, median PFS ~2 mo).	[87,88,89]
	DXd (exatecan derivative)	Topoisomerase I inhibitor (potent, bystander effect)	trastuzumab deruxtecan (HER2-DXd), datopotamab deruxtecan (Trop-2-DXd)	Ongoing PDAC trials; potent in other GI cancers, being tested for HER2/Trop-2 ^+^ PDAC.	[90,91]

## Data Availability

No new data were created or analyzed in this study. Data sharing is not applicable to this article.
